# Lectures on Phthisis Pulmonalis

**Published:** 1885-08

**Authors:** 


**Lectures on Phthisis Pulmonalis,** delivered at the Detroit Medical College, by ERNEST L. SHURLEY, M.D., Professor of Laryngology and Chemical Medicine. Re-printed from the *Medical Age*, of Detroit. Geo. S. Davis, Medical Publisher, Detroit, Mich. 1885.

This is a pamphlet of 117 pages, containing six lectures on consumption. We fail to find anything new in these lectures in regard to this terrible scourge to the human race. They are a carefully prepared digest of the present state of our knowledge, and will be of use to practitioners who desire to read a short, terse and clear exposition of this subject.

